# Azithromycin Mass Treatment for Trachoma Control: Risk Factors for Non-Participation of Children in Two Treatment Rounds

**DOI:** 10.1371/journal.pntd.0001576

**Published:** 2012-03-20

**Authors:** Elizabeth N. Ssemanda, Joshua Levens, Harran Mkocha, Beatriz Munoz, Sheila K. West

**Affiliations:** 1 Dana Center for Preventive Ophthalmology, Wilmer Eye Institute, Johns Hopkins University, Baltimore, Maryland, United States of America; 2 Kongwa Trachoma Project, Kongwa, Tanzania; University of Cambridge, United Kingdom

## Abstract

**Background:**

Persistent non-participation of children in mass drug administration (MDAs) for trachoma may reduce program impact. Risk factors that identify families where participation is a problem or program characteristics that foster non-participation are poorly understood. We examined risk factors for households with at least one child who did not participate in two MDAs compared to households where all children participated in both MDAs.

**Methods/Principal Findings:**

We conducted a case control study in 28 Tanzanian communities. Cases included all 152 households with at least one child who did not participate in the 2008 and 2009 MDAs with azithromycin. Controls consisted of a random sample of 460 households where all children participated in both MDAs. A questionnaire was asked of all families. Random-intercept logistic regression models were used to estimate odds ratios (ORs) and 95% confidence intervals (CIs), control for clustering, and adjust for community size. In total, 140 case households and 452 control households were included in the analyses. Compared to controls, guardians in case households had higher odds of reporting excellent health (OR 4.12 (CI 95% 1.57–10.86)), reporting a burden due to family health (OR 3.15 (95% CI 1.35–7.35)), reduced ability to rely on others for assistance (OR 1.66 (95% CI 1.01–2.75)), being in a two (versus five) days distribution program (OR 3.31 (95% CI 1.68–6.50)) and living in a community with <2 community treatment assistants (CTAs)/1000 residents (OR 2.07 (95% CI 1.04–4.12). Furthermore, case households were more likely to have more children, younger guardians, unfamiliarity with CTAs, and CTAs with more travel time to their assigned households (p-values<0.05).

**Conclusions/Significance:**

Compared to full participation households, households with persistent non-participation had a higher burden of familial responsibility and seemed less connected in the community. Additional distribution days and lessening CTAs' travel time to their furthest assigned households may prevent non-participation.

## Author Summary

The World Health Organization advocates at least three mass drug administrations (MDAs) with antibiotics when the prevalence of follicular trachoma (TF) is greater than 10% in children under age ten. Full child participation is necessary for maximizing the impact of trachoma control programs. The present paper identifies guardian, household, and program risk factors for households with a child who never participated in two annual rounds of MDAs with azithromycin. In comparison to households with full child participation, guardians with at least one child who never participated had a higher burden of familial responsibility, as represented by reporting ill family members, more children, and were younger in age. In addition, guardians of persistent non-participants seemed less well connected in the community, in terms of reliance on others and not knowing who their assigned community treatment assistants (CTAs) were. These guardians were assigned to CTAs who had a wide geographic dispersion of their assigned households. By developing programs with local groups to find and encourage participation in at-risk households, program managers may have the greatest impact on preventing persistent child non-participation. Increasing the number of distribution days and reducing CTAs' travel time may further prevent non-participation.

## Introduction

Trachoma is a leading cause of preventable blindness [1]. Nearly 41 million individuals across the globe are estimated to suffer from active trachoma [2]. Of these, the majority are children from impoverished regions [3], [4]. Virtually all trachoma burden is either concentrated in rural Africa, particularly Ethiopia, Kenya, Niger, Sudan and Tanzania, or parts of Asia [5].

The World Health Organization (WHO) advocates mass drug administration (MDA) in eligible communities as a key component of the Surgery, Antibiotics, Face-washing, Environmental change (SAFE) strategy for treating and preventing trachoma. When a community's prevalence of follicular trachoma (TF) is greater than 10% in children less than age ten years, the WHO supports at least three annual mass drug administrations (MDAs). [6]. In Tanzania, azithromycin is provided to the Ministry of Health free of charge through a donation program from Pfizer Inc and International Trachoma Initiative. For each resident over age one year, a single oral dose of azithromycin at 20 mg/kg up to 1 gram, is recommended, and infants one year and younger are treated with topical tetracycline. Data suggest that endemic communities often require multiple rounds of mass treatment for reducing the prevalence of trachoma [7], [8]. Programs aim for antibiotic coverage goals of at least 80% or more in the entire community [6].

Low treatment coverage with antibiotics in children under ten years is problematic. Young children are a high-risk group for trachoma and infection [9]. Extensive child non-participation in community mass treatments may reduce the effectiveness of trachoma control programs. Untreated children are likely to spread trachoma to other household members and subsequently more individuals in the community [10], [11]. Furthermore, programs squander resources in having to execute additional MDAs when the community treatment coverage is low. Given the WHO recommendations for multiple MDAs in trachoma-endemic communities, characterizing the households with children who never participate is essential. Understanding households with one or more children who never participate in MDAs may help programs develop strategies for avoiding persistent child non-participation.

This study aimed to examine the predisposing and resource risk factors for Tanzanian households with children who never participated in two treatment rounds compared to households where all children participated.

## Methods

The Johns Hopkins Medical Institutional Review Board and the National Institute for Medical Research in Tanzania approved the study protocol. All guardians provided written consent for the study.

### Study Location

We conducted the study in the Kongwa district of Tanzania. Located in the Dodoma region of Tanzania, approximately 250,000 people were residents of Kongwa in 2002 [12]. This study was nested in a larger study [13] of 32 communities which were randomly picked from a list of all communities who met the following criteria: Local government leaders had to provide consent (all communities who were approached did provide consent), and the best-estimated prevalence of trachoma in 2007 was greater than 20% for each community. Due to timing of the MDA, this study was carried out in 28 of the 32 communities.

### Census

As described in [13], prior to each round of MDA, trained research staff completed a census of all households in each community by going to each household and enumerating the residents. Demographic information on each household member was collected at this time. The data were used to develop treatment log books for MDAs. This level of precision was required as part of our research program. In 2008 and 2009, each community received mass treatment.

### Training of Community Treatment Assistants

The Kongwa Trachoma Project (KTP) team trained a group of CTAs, approximately two to six individuals per 1500 persons in each community. Community leaders assisted in identifying persons in the community who would be trusted to deliver MDA, and the KTP staff interviewed and ultimately chose the CTAs. The CTAs received a one-day program discussing trachoma, the disease and consequences, the SAFE strategy, details on azithromycin and possible side effects and how to record them, instructions on how to administer azithromycin by weight to children under one year, and using the height sticks for children greater than one year. If there was doubt as to age one year or less, and the child was below the smallest level of the height stick, the children were weighed. CTAs delivered MDA in their neighborhoods, as would be done in the national Program. We received ethical approval to treat children from one year to 6 months with oral azithromycin, 20 mg/kg, and those under 6 months were treated with topical tetracycline. In addition, the CTAs received training in recording the observed treatment on treatment logs. They also received modest training in asking about vision problems and recognizing trichiasis, in order to keep a record of all persons in the village who had need of further eye care and surgery.

In other districts in Tanzania, there may be modest differences in approaches to MDA; in general the districts provide training to village health workers and community treatment assistants (CTAs) on use of height sticks for treating all residents, with those who are adults (not defined further) receiving 1 gm. Treatment is recorded in log books, and estimated village populations are used to monitor coverage. Two days at least are allotted for MDA, and the CTAs originally, but not since 2006, received monetary incentives.

### Mass Treatment

All communities in the Kongwa district were mass treated on a rolling basis over a period from June to November 2008, and again over the same months in 2009, including communities not in the study. Communities in our study, as part of the larger study were randomly allocated to either a two-day or a five-day distribution program, which began after the census and surveys for the larger study in each community. The June to November time period was chosen because it was after the planting harvest so guardians would be home for mass treatment and to be interviewed. Community treatment assistants offered each resident over six months a single oral dose of azithromycin, 20 mg/kg up to one gram, irrespective of disease status. Oral treatment was directly observed and recorded in a logbook based on the household census. To children less than six months, CTAs gave guardians tetracycline eye ointment to administer topically for four to six weeks. The first dose was instilled but subsequent doses were not directly observed. All communities aimed for treatment coverage greater than or equal to 80% in children under age ten and those in the five day distribution arm were allowed 3 extra treatment days to achieve 90% coverage or greater. At least one member of the KTP staff was in the community each day of MDA, meeting with the CTA and reviewing performance.

Each CTA was assigned a certain number of households for which they were responsible. The CTAs administered treatment to residents at a central location, and if necessary, at the household. CTAs were instructed to review their log books after the first day and if necessary schedule a second central location site or go to each non-participant person's home if necessary and treat them directly. The choice of options was up to the individual CTA, but the goal for the entire community was to achieve at least 80%.

All data on MDA and treatment verification were entered into customized databases.

### Treatment Verification

Standard quality control measures were used to verify coverage. KTP staff went back to a random sample of 5 households per CTA to verify treatment status of all household members. If treatment as recorded in the CTA treatment log was at least 70% concordant with treatment as stated by the family for each member, the CTAs received a small monetary incentive (1,000 TSH or $0.80) per day of work. No CTA was found to be under-performing by this criteria.

### 2009 CTA survey

Data were not routinely collected on characteristics of the CTA. Therefore, for this study each CTA completed a survey on their age, sex and marital status. We also asked about past work experience (e.g. any past MDA experience).

### Identification of Study Population

We used the census and MDA data to identify case and control households with children between six months and nine years at the 2008 census. Our criteria required children to be residents in the households from the 2008 census to the 2009 MDA. Case households included at least one child, between six months to nine years old at the 2008 census, who did not participate in the 2008 and 2009 MDAs. Control households contained children from six months to nine years at the 2008 census who were treated at both MDAs. We did not match or restrict criteria for controls. We interviewed the guardian of the chlld, defined as either the mother or father, or if neither was serving as the guardian, the person in the household who self reported being the guardian. Interviewers surveyed fathers with more than one household in the community only once, and excluded 3 fathers who had already been interviewed.

Our sample size calculation included the following assumptions: alpha = 0.02, beta = 0.20, 2∶1 control to case ratio, prevalence of 30% for most risk factors,and odds ratio of 2.0.We conducted a Bonferroni adjustment for 3 comparisons of the same set of risk factors for this and a companion paper on change in participation. If no correction were applied, we would have had a chance of 0.1426 (14.26%) of finding one or more significant differences in 3 tests. To get an alpha level of 0.05, we lowered the alpha for each test to 0.01667∼0.017. Assuming a non-response/ineligible rate of 15%, the necessary sample size was 330 control households and 165 case households. The adjustment was to make sure we had the correct sample, size, and that we could report at alpha = 0.5, Using simple random sampling, we enrolled a random sample of 460 control households from a larger sample of 5375 control households and all households identified as having at least one child who was a persistent non-participant in MDA. We attempted to contact every case and control household in the 28 communities.

### Data Collection

#### Fieldwork

We trained local field interviewers to administer the risk factor survey and data clerks to enter completed surveys in customized Access databases. At least one to two days prior to data collection, we met with community leaders, explained the study purpose, and requested permission to conduct the study in their community. After obtaining approval, field interviewers surveyed guardians in their homes up to six weeks after the 2009 MDA. Data collection and entry for the risk factor questionnaire took place from mid-July to late October 2009.

#### Risk factors

A formal literature review following extensive search guidelines developed by a senior librarian and the first author was carried out to identify factors associated with MDA for trachoma, lymphatic filariasis, onchocerciasis, and schistosomiasis (these were chosen because they are neglected tropical diseases where MDA is part of the control strategy). The literature resulted in the identification of predisposing and resource factors associated with participation in mass treatment programs. We classified potential risk factors related to the guardian, household, and MDA program as either predisposing or resource factors according to the Andersen Behavioral Model of Health Service Use [14]. Predisposing risk factors were characteristics that predisposed a household not to seek mass treatment (such as guardian age or gender). Conversely, resources risk factors were lack of assets that promoted mass treatment participation.

Our review found the following predisposing risk factors for guardians∶male gender, older age, no formal education (defined as self report of absence of schooling), minority ethnic group membership (defined as self report of tribe other than Gogo or Kaguru), self rated perception of health as excellent (compared to good, fair or poor) the week before the MDA, short-term resident (less than ten years living in the community) and not born in community, non-attendance at the MDA promotional meeting, and recently seeking a traditional healer in the month before the 2009 MDA.

Increasing numbers of children in household, report of ill family members in household (family health burden), household history of child adverse events in 2008 MDA, no perceived trachoma risk in the household were predisposing risk factors for households. We included “traditional belief”, defined as self report of possession by a malevolent spirit (mashetani), as a possible reason for non-participation. Possession with spirits is a common belief in Tanzania, and persons so possessed are instructed by local healers not to mix local medicines with western medicines [15]. Past MDA qualitative reports indicated possession by a malevolent spirit was a reason for not taking azithromycin; thus, we included a question on ever being possessed by a malevolent spirit as a potential predisposing household risk factor. The categories consisted of child and guardian ever possessed, only child ever possessed, only guardian ever possessed, and child and guardian never possessed.

As predisposing factors for programs, we investigated the following: male CTA, guardian's perception of poor CTA performance (defined as rating the CTAs' ability to get azithromycin to the families in the community), longer travel time from the household to the central distribution site, longer travel time between the CTA's household and the furthest assigned household in community and lack of familiarity with his/her assigned CTAs.

We examined resource risk factors for guardians and programs. Resource risk factors for guardians included levels of social interaction, as assessed using a standard questionnaire for contact with family and friends; this was coded as level of contact with other family members (not living in the house but in the community) with everyday versus no/little/some contact. Similarly, for contact with friends, the results were coded as everyday versus no/little/some contact with friends in community. We also evaluated social reliance, with standard questions on inability to rely on others for money and shelter. We categorized inability to rely on others (who do not live with them) for money and shelter: low was no one to rely on for money and shelter, moderate was either someone to rely on for money or someone to rely on for shelter, but not both, and high was someone to rely on for both money and shelter. Finally, the resource risk factors for programs were two (versus five) days distribution and less than two CTAs per 1000 residents. All questions were based on standard questionnaire items from other surveys used in Tanzania, and our questionnaire was vetted through two focus group sessions, using residents from villages not included in the study. Residents from a nearby village in the Kongwa district that did not participate in the study participated in the pilot study, and provided final feed back on the risk factor survey.

There were four sources of risk factors information: the risk factor survey, the MDA log books, the CTA survey and the 2008 census.

### Data Analysis

The primary outcome of interest was a family where at least one child was a continuous non-participant in MDA. We conducted exploratory data analyses, using Pearson's chi-square tests of independent proportions for nominal data, Mann-Whitney tests for ordinal data and t-tests for continuous data. Backwards stepwise logistic regression models assisted in the identification of risk factors with a p-value less than 0.10. One by one, we incorporated each significant risk factor into a random-intercept logistic regression model to evaluate changes in odds ratios and 95% confidence intervals (CIs) and adjust for clustering at the community level. We controlled for community size (small size = bottom 33% communities, medium size = middle 33% communities and large size = top 34% communities) as a confounder and selected the model with the lowest Akaike's information criterion (AIC). We hypothesized that households where all children never participated were different from household where some children never participated. Thus, we performed subgroup analyses with a multinomial model comparing risk factors between: 1) households where every child was a persistent non-participant in both MDAs, 2) households in which some children were persistent non-participants, and 3) households with all children participating in both MDAs. Using this model, the relative risk ratio (RRR) represents the change in the odds of being in the case subgroup versus the control group, where the model can simultaneously estimate the RRR for each case sub-group associated with a one unit change in the independent variable. All analyses were run in STATA ver. 11 (Stata Corp, College Station, Texas).

## Results

In this area, our program offered mass treatment twice, and households with children who never participated in two rounds was quite low, occurring in 2% of 6727 households.

Our study contacted 612 households, 152 with at least one child who was a persistent non-participant and 460 where all children participated in both rounds. According to our 2008 census, contacted households had 2,129 children. The mean community size in the case control study was 1,685 people (standard deviation = 482). Community populations ranged from 750 to 2611 residents in 2008. The mean number of persons per household was five, and the average number of persons under age ten was two children. Of the 612 households, 596 (97%) households completed the risk factor survey.

**Figure 1:**
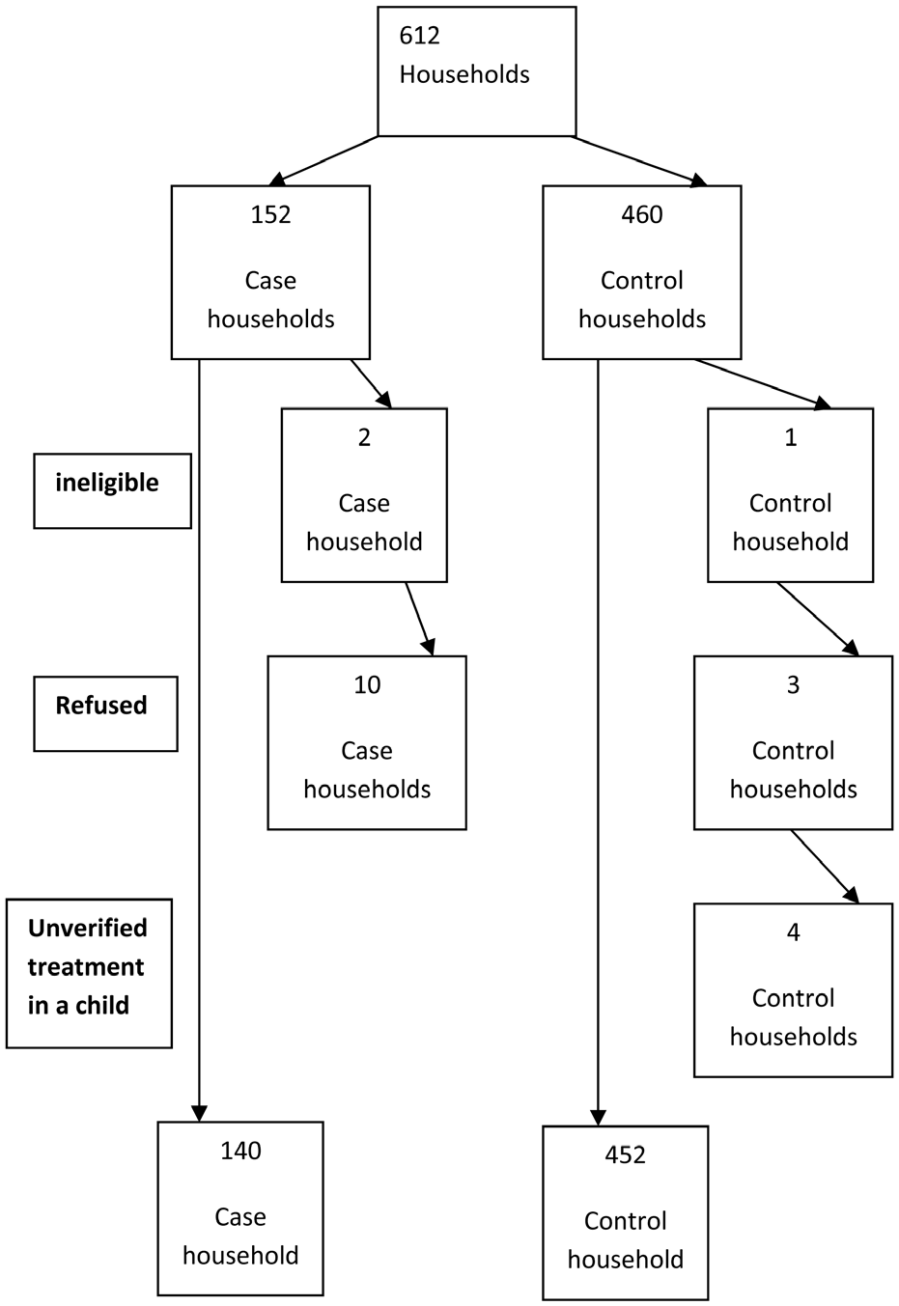
Flow Diagram of participation of case and control households.

Twenty households of the original 612 were not included in the analyses. Three households were ineligible (2 in case households, 1 in the control households). Thirteen households did not respond (10 case households, and 3 control households),, In four control households, we could not be certain if treatment had taken place for each child following treatment verification.. We had no missing information on mass treatment for any child in the surveyed households.(Figure 1) Our study observed no differences in CTA characteristics and guardian demographics between non-response and response households (data not shown).

Some household and program predisposing risk factors were significantly associated with being a household with at least one persistent non-participant (Tables 1 and 2). The risk increased with each additional child in the household of being a household with a persistent non-participant (p<.01). This remained significant after adjusting for other factors (Odds Ratio (OR) = 1.70, 95% Confidence Interval (CI) = 1.39–2.08) (Table 3) Guardians in households with a persistent child non-participant had more than a tenfold odds of not rating the assigned CTAs performance (p = 0.02), but this was also correlated with not knowing their assigned CTAs which was also significantly associated with persistent non-participation (p<0.01). Adjusting for other factors, incorrectly naming or being unable to name their assigned CTAs was associated with an increased risk of persistent non-participation (OR = 1.99 (95% CI = 1.16–3.06) and 5.17 (95% CI = 2.17–12.32), respectively). Compared to households with full child participation, households with persistent child non-participation were more likely to be assigned to CTAs living more than one hour from their furthest assigned household in the community. This relationship persisted after adjustment for multiple factors (OR = 2.58, 95% CI = 1.22–5.44).

**Table 1:** Predisposing factors for persistent child non-participant (Case group) versus full child participation (Control group).

	Cases (n = 140)	Controls (n = 452)	OR*	P-value
	%	%		
**Guardian predisposing factors**				
Each one year decrease in guardian age (mean age shown)	(34)	(35)	1.02	0.08
Guardian never (versus ever) educated	48	45	0.86	0.49
Guardian's health is excellent (versus fair and poor)	92	83	2.24	**0.03**
Guardian residency				
Community born	53	54	1.00	
Long-term resident, born outside the community	11	22	0.50	**0.05**
Short-term resident, born outside the community	36	24	1.54	0.08
Male (versus female) guardian	16	10	1.59	0.14
Traditional healer use				
Did seek healer traditional healer	4	7	1.00	
Did not seek traditional healer	96	93	2.40	0.10
Guardian ethnic group				
Gogo	42	47	1.00	
Kaguru	43	43	0.70	0.21
Other	15	10	1.82	0.09
Guardian attended promotional meeting				
Guardian attended a meeting	14	21	1.00	
Guardian did not attend a meeting	86	79	1.31	0.36
Do not know if attended meeting	0	0.2	NA	NA
**Household predisposing factors**				
Each additional child in the household at 2008 census (mean number of children shown)	(3)	(2)	1.66	**<0.01**
No (versus any) child had itchy or swollen eyes in household	20	16	1.50	0.13
Family possessed with malevolent spirits (mashetani)				
Child and guardian or child only	3	4	1.00	
Guardian only	11	16	0.90	0.86
Not child and not guardian	86	80	1.33	0.61
Family health problems affected (versus did not affect) guardian's ability to perform extra tasks	14	13	1.20	0.57
No (versus any) child with adverse event in 2008 MDA	96	95	1.16	0.77
**Program predisposing factors**				
Travel time from guardian's household to central distribution site&				
0–30 minutes	84	89	1.00	
31–60 minutes	11	8	1.64	0.19
More than 60 minutes	5	3	2.30	0.15
CTA familiarity				
Correctly identifying CTA	39	57	1.00	
Incorrectly identifying CTA	47	39	1.54	0.06
Do not know any CTA	14	4	4.71	**<0.01**
Perception of CTA performance				
Excellent or good	94	98	1.00	
Poor or fair	2	2	1.03	0.97
Do not know	4	0.4	10.98	**0.02**
Male (versus female) CTA	52	52	1.17	0.48
Travel time from CTA's household to furthest assigned household in community				
Less than 30 minutes	29	45	1.00	
31–60 minutes	41	26	2.03	**0.05**
More than 60 minutes	29	29	2.18	**0.04**

CTA: Community treatment assistant.

MDA: Mass drug administration.

OR: Odds ratio.

*: Odds ratios accounted for clustering within communities using random-intercept logistic regression.

NA: Numbers were too small to display odds ratios.

& The sample size number for this odds ratio reduced to 558 households. The CTA visited twenty-one control households and ten case households. Two case households and one control household did not know the time.

**Table 2:** Resource factors for persistent child non-participant (Case group) versus full child participation (Control group).

	Cases (n = 140)	Controls (n = 452)	OR*	P-value
	*%*	*%*		
**Guardian resource factors**				
Frequency of contact with other family				
Every day	42	42	1.00	
Not every day	40	47	1.00	0.99
Do not have other family	18	11	1.83	0.07
Frequency of contact with friends				
Every day	73	82	1.00	
Not every day or do not have friends	27	18	1.65	**0.05**
Social reliance				
High ability to rely on someone for money and shelter	26	37	1.00	
Moderate ability to rely on someone for money and shelter	55	52	1.57	0.07
Low ability to rely on someone for money and shelter	19	11	2.13	**0.02**
**Program resource factors**				
Two (versus five) days distribution	69	46	4.63	**<0.01**
Less than 2 (versus 2 or more) CTAs per 1,000 residents	58	28	3.99	**<0.01**

CTA: Community treatment assistant.

OR: Odds ratio.

*: Odds ratios accounted for clustering within communities using random-intercept logistic regression.

**Table 3:** Model of risk factors for households with at least one persistent child non-participant.

Risk factors	OR*	95% CI	P-value
Reliance on others for money and shelter			
High ability to rely on someone for money and shelter	1.00		
Moderate ability to rely on someone for money and shelter	1.66	(1.01–2.75)	**0.05**
Low ability to rely on someone for money and shelter	1.99	(0.98–4.04)	0.06
Guardian age (per one year decrease)	1.02	(1.00–1.04)	**0.04**
Guardian health reported excellent (versus poor/fair)	4.12	(1.57–10.86)	**<0.01**
Family health burden (versus no burden)	3.15	(1.35–7.35)	**0.01**
Each additional child in the household	1.70	(1.39–2.08)	**<0.001**
Travel time from CTA's household to furthest assigned household in community			
Less than 30 minutes	1.00		
31–60 minutes	1.52	(0.80–2.91)	0.20
More than 60 minutes	2.58	(1.22–5.44)	**0.01**
Two (versus five) days distribution	3.31	(1.68–6.50)	**<0.01**
Less (versus more) than 2 CTAs per 1,000 residents	2.07	(1.04–4.12)	**0.04**
Familiarity with CTA			
Correctly identified at least one CTA	1.00		
Incorrectly identified all CTAs named	1.88	(1.16–3.06)	**0.01**
Did not know any CTAs	5.17	(2.17–12.32)	**<0.01**

CI: Confidence interval.

CTA: Community treatment assistant.

OR: Odds ratio.

*: Odds ratios were adjusted for community size and clustering at community level using random-intercept logistic regression.

A number of guardian and household predisposing factors had no association with household with persistent child non-participation in simple bivariable analyses. Guardian's age, education, perceived health, length of residency, gender, traditional healer use, ethnic group, and attendance at a promotional meeting for mass treatment did not predispose a household to persistent child non-participation. We found no association between persistent non-participation and household predisposing risk factors: reported family health problems, household history of adverse events during the 2008 MDA, and familial possession with malevolent spirits.

Our study found associations between households with a child who never participated and guardian and program resource risk factors (Table 2). Households with a persistent non-participant had low score of social reliance (not being able to ask anyone for money or for a place to live). Compared to households with full child participation, households with persistent child non-participation were more likely to live in a community with a two (versus five) days distribution strategy, and more than a threefold odds of being in a community with less than two CTAs per 1000 residents (p-value<0.01). These factors remained significant when adjusted for multiple factors (Table 3).

After controlling for community size, clustering, and the other variables, our final model identified several independent predisposing and resource risk factors for persistent child non-participation (Table 3). Predisposing factors included younger age and perceived excellent health the week of the 2009 MDA, familial health burden and increasing numbers of children in the family. Resource risk factors included guardians with low scores for social reliance, increased travel time from the assigned CTA's household to the furthest household in the community, less than two CTAs per 1000 residents in the community, and a two days (versus five) days distribution strategy.

Our case and control guardians had some similarities and differences in their response to the general question of the primary reason why parents in the community did not bring their children for treatment (Table 4). In case households, the two most common reasons were travel outside the community during mass treatment and perceived negative side effects from drugs. Control households reported negative side effects from drugs as well, but also felt that general lack of knowledge (stated as “ignorance”) and lack of education on the part of the guardian were explanations for non-participation.

**Table 4:** Primary reason provided for non-participation in MDA by household participation status.*

	Case households with persistent non-participantsn = 140		Control households with full child participationn = 452	
Reasons	n	(%)	n	(%)
Traveling	33	(24)	35	(8)
Negative effects from drug	32	(23)	125	(28)
Lack of education/Ignorance	13	(9)	65	(14)
No notice of treatment	6	(4)	30	(7)
Did not have eye problems	5	(4)	24	(5)
Other	11	(8)	21	(5)
Do not know	40	(29)	152	(34)

*: The chi-square comparison p-value is 0.00.

Of the 140 households that completed the risk factor survey, 54 (40%) were households in which all children never participated in both MDAs. The remaining 86 (60%) households contained some children who had participated in one or both rounds as well children who were non-participants in both rounds. We hypothesized differences between these two subgroups, compared to households where all children participated. Common factors for both groups were program predisposing and resource factors: being in a two days distribution program and not being familiar with any assigned CTA (Table 5). However, compared to households with full child participation, households where all children never participated both times had healthier guardians, expressed a family health burden, and were in a program with less than two CTAs per 1000 residents.

**Table 5:** Multinomial logistic model comparing risk factors for case subgroups with control group.*

	All children are cases			Some children are cases		
Risk factors	RRR	95% CI	P-value	RRR	95% CI	P-value
Less able to rely on others for money and shelter						
High ability to rely on someone for money and shelter	1.00			1.00		
Moderate ability to rely on someone for money and shelter	1.52	(0.75–3.08)	0.25	1.69	(0.94–3.06)	0.08
Low ability to rely on someone for money and shelter	2.11	(0.73–6.06)	0.17	2.11	(0.92–4.82)	0.08
Each one year decrease in guardian age	1.02	(0.98–1.05)	0.33	1.03	(1.00–1.05)	**0.03**
Guardian's health is excellent (versus poor/fair)	6.84	(1.90–24.59)	**<0.01**	3.12	(0.84–11.53)	0.09
Family health burden (versus no burden)	5.23	(1.87–14.65)	**<0.01**	2.33	(0.74–7.32)	0.15
Each additional child in the household	0.92	(0.62–1.38)	0.69	2.31	(1.86–2.87)	**<0.01**
Travel time from CTA's household to furthest household in community						
Less than 30 minutes	1.00			1.00		
31–60 minutes	1.48	(0.68–3.22)	0.32	1.46	(0.72–2.96)	0.29
More than 60 minutes	1.31	(0.47–3.67)	0.60	4.79	(1.99–11.51)	**<0.01**
Two (versus five) days distribution	2.03	(1.07–3.85)	**0.03**	4.29	(2.14–8.60)	**<0.01**
Less (versus more) than 2 CTAs for 1,000 residents	2.34	(1.11–4.95)	**0.03**	1.69	(0.89–3.20)	0.11
CTA familiarity						
Correctly identified at least one CTA	1.00			1.00		
Incorrectly identified CTA	2.15	(1.09–4.26)	**0.03**	1.88	(1.06–3.34)	**0.03**
Did not know any CTAs	9.01	(3.33–24.34)	**<0.01**	3.21	(1.12–9.17)	**0.03**

CI: Confidence interval.

CTA: Community treatment assistant.

RRR: Relative risk ratio represents the change in the odds of being in the case subgroup versus the control group associated with a one unit change in the independent variable.

*: Relative risk ratios were adjusted for community size and models used robust standard error estimates.

Households where some children participated and others did not in both rounds had other risk factors. In comparison to households with all children treated both times, each additional child in the household increased the risk of having a household where some (but not all) children were persistent non-participants. In addition, these households had younger guardians, and were assigned CTAs living more than one hour from the furthest assigned household.

## Discussion

We have previously shown that treatment non-participation clusters within families in these communities [16]. This study builds on our previous research and suggests that several predisposing and resource factors at the guardian, household, and program level are associated with child non-participation in families. Findings of equal importance are some predisposing and resource factors which did not seem to be related to persistent non-participation in children. These are discussed below.

### Guardians

Guardians exert a strong influence on their children's healthcare. It is therefore important to ensure that trachoma control programs providing mass treatment address guardian concerns and barriers. Identifying guardian characteristics of households' with persistent child non-participation may help programs target households at-risk.

Among the possible guardian predisposing and resource risk factors studied, younger guardian age, perceived excellent health, and decreased ability to rely on others were useful markers of households with persistent child non-participation. Similar to our study, other child health services have found younger guardian age is a risk factor for lower use of child health services [17]. This variable was more important for households where not all children were persistent non participants, which suggests the difficulty young guardians have in bringing all children to MDA.

Guardians in households with persistent child non-participation perceived their health as better during the week of mass treatment compared to guardians in households with full child participation. This result is comparable to another program that found people who were healthy tended to not participate in mass treatment [18]. Guardians in households with full child participation may have been less healthy and thus more likely to take their children for MDA because they themselves also wanted to be treated. Also, those who report being healthy were more likely to be guardians of households where all children did not participate, suggesting that there was no perceived need for treatment or low priority was given to participation. We found no difference between the case and control households in perceived risk of trachoma in their children, suggesting that general self-perception of health may be more important than messages about trachoma.

Social reliance or the ability to rely on other individuals for money or a place to live was an important guardian resource that households with persistent child non-participation lacked. That ability to rely on others is a key part of kinship systems, systems that continue to thrive in Tanzania [19]. A high degree of reciprocal exchange of goods and services in these systems exists, and it is through this sharing of resources that the groups thrive. Social networks provide an informal social security; research has demonstrated a positive association between larger strong social networks and well-being in low-income countries [19]. Guardians who could not rely on others for money or shelter were likely not as deeply supported as were other guardians in the community. The association was strongest for guardians of households where at least some children participated in one or both rounds. For these guardians, getting help transporting all their children to the central distribution site for treatment in both MDAs was possibly more difficult. Thus, distribution strategies that target marginalized households - guardians who cannot rely on others for money or a place to live- and encourage healthy guardians to bring their children for treatment as a way of keeping them and their family healthy, may prevent persistent child non-participation.

### Household

An understanding of household barriers may also assist programs in recognizing households at-risk for persistent child non-participation. We found that reported family health problems severe enough to interfere with daily tasks was a strong risk factor for households with persistent child non-participation. This was especially the case for households where no children participated. The family health burden originated from family members (e.g. husband, grandmother) not from the guardian, as the guardian perceived him/herself as healthy. Research has linked a stressed guardian to lower use of child health services [20]. Guardians under stress, potentially caused by a health burden within the family, must contend with many hardships that compete with MDA.

Multiple young children in the household were a characteristic of households with some children who never participated. Our results were consistent with a previous study that examined households factors associated with azithromycin coverage after a single round [21], which also found that having more children residing in the household adversely influenced household coverage. Guardians may find the logistics of bringing all children in the household to a treatment center difficult if the number in a household is large. Additional studies have verified that mothers with multiple children are at high risk for having under-vaccinated children and using fewer primary care services for children [20], [22], [23]. As noted, these households tend to have younger guardians with less ability to rely on others as well, providing an image of over-burdened young guardians who tend not to participate.

Social mobilization programs, involving local groups, have been useful in targeting marginalized households at-risk for non-participation in child health services [24]. In Dhaka, Bangladesh, an intervention package including a supportive group for social mobilization was valuable in moving from 43% children fully immunized to 99% children fully immunized. Since we now know characteristics of households with children who persistently do not participate, we could use a similar strategy to identify and assist households with many young children who have less ability to rely on others, or describe having a family member with an illness.

### Program

Modifying some treatment program characteristics related to visibility, access and organization might reduce persistent child non-participation. Inability to name the CTAs was one program factor in our study that could be targeted. Other mass treatment programs have observed non-familiarity with CTAs as a risk factor for individual non-participation [25], [26]. In these MDAs, community members did not trust CTAs because they were unknown and not part of their community. However, as most CTAs in our study were from the community, this is not likely the problem and may reflect the fact that if the household did not participate, they did not meet the CTA. However, the CTA was supposed to travel to the household to offer MDA, and this finding suggests that this was not always the case. Future MDAs should ensure that in the case of non-participation the CTA visit the household.

One program feature was related to less accessibility. Community treatment assistants living more than one hour from the furthest assigned household were characteristic of households with persistent child non-participation. Ivermectin MDAs for onchocerciasis also observed further distance from the CTA's household to the furthest assigned households was an issue. The CTAs working within one km were more likely to attain 90% treatment coverage in the community [27]. With greater travel time in a community, CTAs have less motivation to return several times to treat non-respondents, especially if there are only a few in a household that otherwise participated. This supposition is supported by our finding that this risk factor is more important for households where some, but not all, children were persistent non-participants.

Programs seeking to stop persistent child non-participation could also address accessibility by increasing the number of distribution days and improve organization by increasing the number of CTAs per 1000 residents. In our study, supplemental treatment distribution days appeared to provide parents with more flexibility; Guardians could bring their children for treatment on days that were convenient for them. Past research in child immunization programs verified that shorter distribution time was associated with non-participation [28], [29]. Modifications in the schedule allowed more guardians to attend a location, particularly working mothers. In addition, more assigned CTAs at the central distribution site cut the treatment lines, helped the drug administration process run more efficiently, and allowed CTAs time to visit households on more than one occasion. However, case and control households both resided in communities that had two and five days distribution programs so just increasing days alone is not the only factor. Given that the research provided a small incentive for CTA time doing MDAs, the cost per additional coverage needs to be evaluated.

Factors related to the MDA delivery system (good training, community government support, CTA incentives) are liable to influence the effectiveness of treatment assistants positively, and this program contained all of these elements. An experienced non-government organization, KTP, supervised CTAs during the course of the MDA through daily observations. Furthermore, the community leadership recommended and supported CTAs. In addition, most CTAs were residents in their communities, so other residents in mass treatment programs would likely be familiar with their CTAs, even if they did not know they had taken on that responsibility. Following treatment verification of their work quality, the program offered CTAs an incentive for completing high coverage. Thus, we could not measure the effect of lack of incentives, or CTAs chosen by other mechanisms or lack of supervision as possible additional program factors.

Increasing distribution time and number of CTAs in programs alone may not always result in improved performance, as the other training components for CTAs, CTA incentives, and support from local community are likely important overall factors as well. Nevertheless, with such additional factors in mind, programs like ours that have these elements in place should consider allotting funds for increased distribution time and more local personnel to improve participation.

Finally, programs need specific interventions for households where all children never participated and households where some children never participated. Our study found each group had guardians strained in different ways. Strategies for encouraging households where some children never participated could include providing CTAs with bikes to travel to families, and working with local groups to reach out to younger guardians and those with multiple young children. For households where all children never participated, CTAs could work with local groups to identify households with guardians caring for sick family members, develop a protocol for “mop-up” treatment, and assist these guardians in getting their children treated. Hiring more than two CTAs for every 1000 residents may also enable the program to reach households where children never participated.

### Study Strengths

The strengths in this study include minimal misclassification of cases and controls due to direct observation and recording of treatment, and the high participation among cases and controls. Treatment was directly observed by the CTA at the time of distribution. CTAs were spot-checked by KTP staff during the implementation of MDA, and treatment verification was carried out to ensure that records were maintained correctly. Therefore, we are confident that reporting errors were rare. Community treatment assistants could have over-reported compliance. However, treatment verification for the 2008 and 2009 were exceptional. Our study found misclassification in less than 1% of households in our study.

We had very high response rate to the survey, 92% case households and 98% of control households. We found no differences in any CTA and census characteristics for case response households and case non-response households. Thus, we were confident that the risk factors found in our study have minimal bias due to non-response.

### Study Limitations

Case control designs have limitations, notably the problem of recall bias. We retrospectively collected guardian time-dependent risk factor data three to six weeks after the 2009 mass treatment. Data may not be accurate if parents did not recall the information correctly, such as the state of their health or the other members of the family. We attempted to improve guardian recall by providing guardians with the exact dates of mass treatment during the field interview. Guardians were prompted with the number of weeks since mass treatment for mass treatment questions in the survey. Since recall bias might be in any direction it is difficult to predict how this might impact findings. Second, our study may have missed additional important factors, especially as related to the first, 2008, MDA. We did not ask about factors related to the first MDA as it was over a year ago, but instead factors related to the second MDA. However, conducting a prospective study, with data collection immediately prior to each mass treatment, was not possible.

We also recognize that the non –participation studied here is in the context of a generally high participation rate using CTA's that were motivated and incentivized. Thus, in other settings with lower coverage or different MDA program design, program factors may emerge as even more important than what we observed. Nevertheless, the guardian and program factors we did find are likely to have good generalizability to other settings similar to the communities sampled in this study.

### Conclusion

Trachoma remains the most common infectious cause of blindness and is likely to remain so in the near future. Although treatment participation for trachoma was high in this study, at both rounds, there were households with children who never participated. We identified predisposing and resource risk factors for these households that programs may address with more effort. Program designs should consider targeting marginalized households (e.g. multiple children in household, family health burden) through social mobilization programs. By increasing the number of distribution days to improve program access and by increasing the number of treatment assistants to help program organization, in addition to proper training, incentives, and record keeping, the program may decrease persistent child non-participation. Moreover, partnerships and buy-in with community leadership and liaison groups will no doubt be vital for the long-term successful trachoma control program. We dispelled the notion that households were passive recipients or apathetic non-participants of mass treatment programs. In actuality, households judge the treatment's value and relevance against their children's needs and factors related to the guardian, household, and program. In their design, programs can target these risk factors. The recognition of risk factors for households with persistent child non-participation is a critical step for implementing programs encouraging full child participation over time.
